# Using an engineered glutamate-gated chloride channel to silence sensory neurons and treat neuropathic pain at the source

**DOI:** 10.1093/brain/awx201

**Published:** 2017-08-19

**Authors:** Greg A Weir, Steven J Middleton, Alex J Clark, Tarun Daniel, Nikita Khovanov, Stephen B McMahon, David L Bennett

**Affiliations:** 1 Nuffield Department of Clinical Neurosciences, University of Oxford, Oxford, UK; 2 Wolfson CARD, King’s College London, London, UK

**Keywords:** neuropathic pain, ion channels, sensory systems, peripheral nerve injury, gene therapy

## Abstract

**See Basbaum (doi:10.1093/brain/awx227) for a scientific commentary on this article**.

Peripheral neuropathic pain arises as a consequence of injury to sensory neurons; the development of ectopic activity in these neurons is thought to be critical for the induction and maintenance of such pain. Local anaesthetics and anti-epileptic drugs can suppress hyperexcitability; however, these drugs are complicated by unwanted effects on motor, central nervous system and cardiac function, and alternative more selective treatments to suppress hyperexcitability are therefore required. Here we show that a glutamate-gated chloride channel modified to be activated by low doses of ivermectin (but not glutamate) is highly effective in silencing sensory neurons and reversing neuropathic pain-related hypersensitivity. Activation of the glutamate-gated chloride channel expressed in either rodent or human induced pluripotent stem cell-derived sensory neurons *in vitro* potently inhibited their response to both electrical and algogenic stimuli. We have shown that silencing is achieved both at nerve terminals and the soma and is independent of membrane hyperpolarization and instead likely mediated by lowering of the membrane resistance. Using intrathecal adeno-associated virus serotype 9-based delivery, the glutamate-gated chloride channel was successfully targeted to mouse sensory neurons *in vivo*, resulting in high level and long-lasting expression of the channel selectively in sensory neurons. This enabled reproducible and reversible modulation of thermal and mechanical pain thresholds *in vivo*; analgesia was observed for 3 days after a single systemic dose of ivermectin. We did not observe any motor or proprioceptive deficits and noted no reduction in cutaneous afferent innervation or upregulation of the injury marker ATF3 following prolonged glutamate-gated chloride channel expression. Established mechanical and cold pain-related hypersensitivity generated by the spared nerve injury model of neuropathic pain was reversed by ivermectin treatment. The efficacy of ivermectin in ameliorating behavioural hypersensitivity was mirrored at the cellular level by a cessation of ectopic activity in sensory neurons. These findings demonstrate the importance of aberrant afferent input in the maintenance of neuropathic pain and the potential for targeted chemogenetic silencing as a new treatment modality in neuropathic pain.

## Teaser

Following nerve injury, sensory neurons become hyperexcitable and this is a key driver of neuropathic pain. Weir *et al.* develop a gene therapy approach that silences sensory neurons and reversibly normalizes pain thresholds in animal models of neuropathic pain. This new approach has significant translational potential.

## Introduction

Neuropathic pain arises as a consequence of a lesion or disease of the somatosensory nervous system and affects 7–10% of the general population (Colloca *et al.*, 2017). Many of the most common causes of neuropathic pain such as post-herpetic neuralgia, diabetic polyneuropathy and traumatic nerve injury, relate to injury to sensory neurons within the peripheral nervous system. A key pathophysiological driver for the induction and maintenance of neuropathic pain in such conditions is the development of hyperexcitability of sensory neurons, which develop ectopic activity (Wall and Gutnick, 1974; Wall and Devor, 1983; Kajander and Bennett, 1992; Boucher *et al.*, 2000; Wu *et al.*, 2001) and an abnormal response to natural stimuli (Shim *et al.*, 2005; Smith *et al.*, 2013). Such abnormalities have been demonstrated in multiple animal models of peripheral nerve injury (Kajander and Bennett, 1992; Boucher *et al.*, 2000; Wu *et al.*, 2001) as well as in patients suffering from peripheral neuropathic pain through the use of microneurography (Serra *et al.*, 2012). Ectopic activity develops in small diameter unmyelinated and large diameter myelinated sensory axons, and arises at the site of the injured axon and at the level of the cell body (Wall and Devor, 1983). This hyperexcitability occurs due to profound changes in the expression as well as altered trafficking and post-translational modifications of ion channels within sensory neurons (Waxman and Zamponi, 2014).

Studies on patients using local anaesthetic blocks suggest that abnormal afferent input from sensory neurons is required for the maintenance of peripheral neuropathic pain (Haroutounian *et al.*, 2014; Vaso *et al.*, 2014). Systemic lidocaine reduces ectopic activity in animal models of nerve injury (Devor *et al.*, 1992); however, dose escalation in humans is limited by motor, cardiac and CNS side effects. Similarly, the oral analgesics currently available that target aberrant afferent input have limited efficacy and poor tolerability (Finnerup *et al.*, 2015).

Chemogenetics provides opportunities for a novel treatment approach to neuropathic pain by silencing aberrant primary afferent input. This technique enables precise control of neuronal activity through expression of receptors/ion channels that have been engineered to be solely activated by a non-toxic drug (Sternson and Roth, 2014). This allows for temporal and spatial control of activity, determined by the pattern of expression of the receptor and timing of drug administration. From a translational perspective, a key attribute of the chemogenetic approach is that it enables non-invasive and chronic modulation of neuronal activity. A heteromeric glutamate-gated chloride channel (GluCl v1.0) modified to respond to low doses of ivermectin but not glutamate, has previously been shown to silence activity of mammalian CNS neurons *in vivo* for several days following a single dose of ivermectin (Lerchner *et al.*, 2007; Lin *et al.*, 2011)*.* Effectiveness was limited by difficulties of ivermectin crossing the blood–brain barrier and suboptimal β-subunit trafficking (Lerchner *et al.*, 2007). We therefore used a recently optimized version of GluCl (GluCl v2.0), wherein ivermectin sensitivity and effective trafficking of the β-subunit have been increased (Frazier *et al.*, 2013) with the aim of silencing peripheral sensory neurons.

## Materials and methods

### Animals

Young (4–8-week-old) wild-type C57BL/6 mice (University of Oxford Breeding Unit) of both genders were used for *in vitro* culture experiments including electrophysiology, somal Ca^2+^ imaging and immunocytochemistry. Adult male (6–8 weeks old at time of intrathecal surgery) C57BL/6 mice were used for all behavioural experiments. For microfluidic experiments, embryonic Day 16 Sprague-Dawley rat embryos (Charles River) were used. Animals were group-housed in specific pathogen-free conditions on a 12:12 h light–dark cycle at 23 ± 1°C with free access to food and water. All behavioural testing was performed during the light phase. Animals were assigned to treatment groups by randomly drawing them from their housing cages and rehoused post-treatment in mixed cages. All procedures complied with the UK Animals (Scientific Procedures) Act (1986) and were performed under a UK Home Office Project Licence in accordance with University of Oxford Policy on the Use of Animals in Scientific Research. This study conforms to the ARRIVE guidelines (Kilkenny *et al.*, 2013).

### Plasmids and AAV production

Control (GFP) plasmid was pAcGFP1-C1 (Clonetech Laboratories). Constructs for GluCl v2.0 (Opt α-GluClv2.0 and Opt β-GluClv2.0) were a gift from Henry Lester (Addgene plasmids 47387 and 47542) (Frazier *et al.*, 2013). The yellow fluorescence protein (YFP) sequence of α-GluClv2.0 was replaced by the sequence for monomeric Cerulean (mCerulean; Rizzo *et al.*, 2004) (gift from Dave Piston, Addgene plasmid 15214) using EcoRI cloning sites. α- and β-GluCl transgenes were excised and inserted separately into a pAAV (adeno-associated virus) packaging plasmid with the use of BamHI/NotI (β) and HindIII/NotI (α) restriction sites. The packaging plasmid contained the inverted terminal repeats of AAV2 flanking a 1.8 kb CAG promoter, the transgene and a bovine growth hormone (bGH) polyadenylation signal. Constructs were commercially packaged and serotyped with the AAV9 capsid protein by Penn Vector Core in the Gene Therapy Program of the University of Pennsylvania to a final titre of 2.4 × 10^13^ gene copies (GC)/ml (GluClβ-AAV) and 1.5 × 10^13^ GC/ml (GluClα-AAV).

### Surgical procedures

Intrathecal delivery of AAV was performed on 6–8-week-old mice (18–25 g) under 2% inhaled isoflurane anaesthesia. The dura was exposed by careful dissection of soft tissue between T10 and T11 vertebrae and punctured with a 30 G needle, before a small cannula (polyetrafluoroethylene tubing, 0.008” outer diameter × 0.004” internal diameter, Braintree Scientific) was inserted 1 cm caudally into the subarachnoid space. Eight microlitres of AAV [4 μl of neat GluClβ-AAV with 4 μl of neat GluClα-AAV, or phosphate-buffered saline (PBS)] was then infused at a rate of 1 μl/min. Following a 1-min rest period to limit backflow, the cannula was slowly removed and the animal recovered with the administration of local (2 mg/kg Marcain, AstraZeneca) and systemic (5 mg/kg Rimadyl, Pfizer) postoperative analgesia. The time between intrathecal infusion and animal sacrifice to assess viral transduction or the commencement of behavioural analysis was at least 3 weeks.

For the spared nerve injury (SNI) model we ligated and transected the tibial and common peroneal branches of the sciatic nerve, leaving the sural nerve intact (Decosterd and Woolf, 2000). Animals received postoperative analgesia as detailed above and were assessed behaviourally from 3 days post-SNI in addition to being monitored for self-mutilation (autotomy) daily. Autotomy of two or more nails/toes led us to sacrifice the animal. Any animals that did not show a 50% reduction in mechanical thresholds by 7 days post-SNI were excluded from further assessment and were not included in the final analysis. The presence of autotomy or the lack of mechanical hypersensitivity led us to exclude 2/30 animals. For whole-cell patch clamp experiments L3, L4 and L5 dorsal root ganglia (DRG) were pooled from animals that had undergone SNI surgery 3–5 weeks previously to generate DRG cell cultures (see below).

### Ivermectin intraperitoneal injections

A 1% sterile solution of ivermectin (Noromectin®, Norbrook Laboratories) was diluted to 0.5% in sterile propylene glycol, prior to intraperitoneal (i.p.) injection at 1 μl/g animal body weight.

### Behavioural testing

All behavioural studies were performed by an experimenter blind to treatment group and at a consistent time of day. Sample sizes were chosen based on similar experiments in the literature (thermal place preference experiments; Bautista *et al.*, 2007), or calculated using standard power calculations based on our own previous studies (statistical power of 80% and α-value < 0.05). For assessment of mechanical and thermal thresholds the minimum group size required to show a 25% change was 9 and the group size required to detect a 25% rescue in mechanical sensitivity following SNI was 8. Application of von Frey hair filaments (Stoelting) to the plantar surface of the hind paw using the ‘up-down’ method was used to assess mechanical sensitivity. Prior to testing, mice were acclimatized for 1 h on an elevated wire mesh floor and housed in 8 × 5 × 10-cm partitions. The 50% paw-withdrawal threshold was calculated as per Dixon (1980) on three different days and the average taken as the baseline value. Thermal thresholds were assessed using an infrared light source applied to the plantar surface of each hind paw (Hargreaves test). Latency to withdraw was tested three times per paw and averaged. All baseline data represent the average score of three separate days of testing. Except in the case of nerve injury, all sensory threshold data represent the average of right and left hind paws. Noxious mechanosensation was assessed by the pinprick test described previously (Arcourt *et al.*, 2017). Mice were housed and acclimatized similarly to the von Frey test. A pinprick stimulus was applied to the plantar surface of the hind paw by a dissecting pin attached to a 1 g von Frey filament. Latency to withdraw was recorded by a Sony Xperia phone at 120 fps (8.33 ms per frame) and analysed by an investigator blind to treatment groups. Three measurements were taken for each hind paw per trial and averaged. Plotted latency represents the average of both paws. Baseline data represent the average of two trials performed on separate days.

Motor performance was assessed by the rotarod performance test (Ugo Basile, 47600). Mice were scored for their latency to fall from a horizontal cylinder rotating at 32 rpm. Animals were acclimatized to the equipment several days prior to testing by placing them on the cylinder; first while it was stationary and then while rotating. Following acclimatization, two baseline measurements were taken on separate days and averaged to generate baseline data. Each data point represents one trial performed on a given day. The Beam test (Carter *et al.*, 2001) apparatus consisted of a 1-m long horizontal beam of 12-mm width suspended from the ground. Mice were placed at one end of the beam next to a light source and walked across the beam towards a darkened box. Trials were recorded once per day and video footage was used by an investigator blind to treatment groups, to assess number of mistakes (defined as missed hind paw placements or a hop). Mice were acclimatized to the apparatus prior to testing for 10 min. Baseline data were generated from the average of two trials on separate days.

Thermal place preference was performed with the use of two chambers with aluminium plates [in cm, 25 (w) × 37 (d) × 47 (h); Ugo Basile] as the base, connected by a short passage. One plate was maintained at room temperature (22°C) and the other was set to 16°C. Assigning temperatures to each plate was performed at random to prevent animal learning. The percentage time spent at each temperature was live-scored over a 10-min period. Animals were acclimatized to the equipment several days prior to testing by placing them on the plates at room temperature for 10 min. Following acclimatization, two baseline measurements were performed on separate days and averaged to generate baseline data.

### Immunohistochemistry

For tissue collection, animals were deeply anaesthetized with pentobarbital and perfused through the vascular system with ice-cold 0.9% NaCl solution until tissues were sufficiently cleared of blood. L4 DRGs, nodose ganglia, superior cervical ganglia, lumbar sympathetic chain, hind paw glabrous skin and peripheral nerves were collected and post-fixed for 1–2 h in 4% paraformaldehyde (PFA). Lumbar spinal cord and brain tissue were collected simultaneously and post-fixed in 4% PFA overnight. Post-fixation, all tissue was stored in 30% sucrose for a minimum of 48 h before being embedded and sectioned by a cryostat. DRG, skin and nerve were sectioned at 15 μM, while brain and spinal cord were sectioned at 30 μM. Cell cultures were fixed at room temperature for 10 min in 4% PFA and from there on treated similarly to tissue sections.

Samples were incubated at room temperature for 1 h in blocking solution (PBS + 0.3% Triton™ X-100 + 10% normal goat serum), before overnight incubation with primary antibody at 4°C. Antibodies used in this study were as follows: NeuN (1:500, rabbit, ab177487, Abcam), GFP (1:500, chicken, ab13970, Abcam), NF200 (1:1000, rabbit, ABN76, Merck Millipore), CGRP (1:1000, rabbit, T-4032.0050, Peninsula Laboratories) IB4 (1:50, streptavidin conjugated, L2140, Sigma-Aldrich), β-III-tubulin (1:500, rabbit, D71G9, Cell Signalling), parvalbumin (1:200, guinea pig, Af1000, Frontier Institute), PGP 9.5 (1:200, rabbit, 516-3344, Zytomed), ATF-3 (1:1000, rabbit, sc-81189, Santa Cruz) and BRN3A (1:100, rabbit, Ab5945, Millipore). Samples were then incubated with corresponding fluorophore conjugated secondary antibodies (1:1000, Alexa Fluor® 488, 546 and 1:500 Pacific Blue Dye, Thermo Fischer Scientific), diluted in blocking solution for 2 h at room temperature. Samples were mounted and viewed by a Zeiss LSM-700 confocal microscope and ZEN software. Image analysis was performed using Fuji/ImageJ (NIH). For image quantification, data were averaged from at least three sections per tissue and sampled from at least three animals per group. Only neurons with a clear nucleus were included in the analysis of DRG cell size distribution.

### Dorsal root ganglia neuron culture and electroporation

DRG were rapidly excised (Sleigh *et al.*, 2016) and enzymatically digested at 37°C for 80 min in dispase type II (4.7 mg/ml) plus collagenase type II (4 mg/ml) (Worthington Biochemical). DRG from all levels were used except for experiments examining biophysical properties following SNI wherein only L3, L4 and L5 tissues were harvested. Mechanically dissociated cells were plated onto laminin/poly-d-lysine (BD Biosciences) treated coverslips in complete Neurobasal® medium [Neurobasal® media supplemented with 2% (v/v) B27 and 1% (v/v) penicillin streptomycin (ThermoFisher Scientific)]. Mouse nerve growth factor (50 ng/ml; NGF, PeproTech) and 10 ng/ml glial-derived neurotrophic factor (GDNF, PeproTech) were added to the media and cultures used for experiments 24–72 h later. For cultures generated from GluCl AAV animals, neurons were plated and maintained in DMEM-F12 with 10% foetal calf serum (ThermoFisher Scientific) instead of complete Neurobasal® medium and used for experiments 4–24 h after plating. Electroporation was performed prior to plating using the Neon® transfection system (ThermoFisher Scientific). Dissociated neurons were resuspended at 5 × 10^6^ cells/ml in 10 µl Buffer R with 1 µg of total plasmid DNA. The electrical protocol applied was three 1500-V pulses of 10-ms duration. After electroporation, cells were immediately plated as described above and used 24–72 h later, when expression of the plasmid DNA was maximal.

### Human embryonic kidney cell culture

HEK 293 cells were routinely subcultured in Dulbecco’s modified Eagle medium (DMEM; ThermoFisher Scientific) with 10% foetal calf serum. Cells were plated in six-well dishes 24 h prior to transfection with Lipofectamine™ 3000 (ThermoFisher Scientific) and plasmid DNA (total of 1 μg DNA). Twenty-four hours post-transfection, cells were replated onto coverslips at low density, before being used for patch clamp experiments 24–48 h later.

### Derivation of sensory neurons from induced pluripotent stem cells

The control induced pluripotent stem cell (iPSC) line AD2-1 was derived from commercial fibroblasts (Lonza, CC-2511) and reprogrammed using the CytoTune® iPS Reprogramming Kit (ThermoFisher Scientific). Quality control checks of this line included: tests for Sendai virus clearance, fluorescence-activated cell sorting (FACS) for pluripotency markers, genomic integrity checks and embryoid body tri-lineage differentiation experiments. For differentiation, iPSCs were passaged onto Matrigel®-coated six-well plates using TrypLE express (ThermoFisher Scientific) and maintained in mTeSR1 supplemented with 10 μM ROCK inhibitor (ScienCell). Twenty-four hours after plating, the medium was exchanged to mouse embryonic fibroblast (MEF) conditioned medium (ScienCell) supplemented with 10 ng/ml human recombinant FGF2. Cells were allowed to expand on MEF-conditioned medium until ∼50% confluent, at which time differentiation was started according to Chambers *et al.* (2012). Briefly, medium was exchanged to knockout serum replacement (KSR) medium containing; knockout-DMEM, 15% knockout-serum replacement, 1% GlutaMAX™, 1% non-essential amino acids, 100 μM β-mercaptoethanol, 1% antibiotic/antimycotic (ThermoFisher Scientific), supplemented with the SMAD inhibitors SB431542 (Sigma, 10 μM) and LDN-193189 (Stratech, 100 nM). The medium was gradually transitioned from KSR medium to N2 medium (Neurobasal® medium, 2% B27 supplement, 1% N2 supplement, 1% GlutaMAX™, 1% antibiotic/antimycotic) (ThermoFisher Scientific) over an 11-day period. On Day 2, the small molecules CHIR99021 (Apollo Scientific, 3 μM), SU5402 (R&D Systems, 10 μM) and DAPT (Sigma, 10 μM) were also added. SMAD inhibitors were removed from the media from Day 6 onwards. On Day 11, the now immature neurons were replated onto Matrigel®-coated coverslips (25 000 cells per 13 mm coverslip) in 100% N2 medium containing human recombinant NGF, GDNF, BDNF, NT3 (all at 25 ng/ml, PeproTech) and 10 μM ROCK inhibitor. CHIR99021 (3 μM) was included in the medium until Day 14, and laminin (1 μg/ml, ThermoFisher Scientific) was supplemented into the medium from Day 20 onwards. Medium changes were performed twice weekly after replating. Cytosine β-d-arabinofuranoside (AraC, 2 μM, Sigma) was included in the medium for 24 h following replating to remove the few non-neuronal dividing cells remaining in the culture. This differentiation resulted in a pure neuronal culture with extensive arborized neurites by 2–3 weeks after the end of the small inhibitor stage. Neurons were matured for 4 weeks before addition of AAV (GluClβ-YFP-AAV with or without GluClα-mCerulean-AAV) at a multiplicity of infection (MOI) of 10^5^. All patch clamp and immunocytochemistry experiments were performed 1–3 weeks post-virus treatment.

### Electrophysiology

Whole-cell patch clamp recordings were performed at room temperature (22°C) using an Axopatch 200B amplifier and Digidata 1550 acquisition system (Molecular Devices). GluCl^+^ DRGs were defined by the presence of YFP and detected with an Olympus microscope with an inbuilt GFP filter set (470/40× excitation filter, dichroic LP 495 mirror and 525/50 emission filter). Data were low-pass filtered at 2 kHz and sampled at 10 kHz. Series resistance was compensated 60–80% to reduce voltage errors. Patch pipettes of 2–6 MΩ tip resistance were pulled from filamental borosilicate glass capillaries (1.5 mm outer diameter, 0.84 mm inner diameter; World Precision Instruments) and filled with internal solution containing (mM): 100 K-gluconate, 28 KCl, 1 MgCl_2_, 5 MgATP, 10 HEPES, and 0.5 EGTA; pH was adjusted to 7.3 with KOH and osmolarity set to 305 mOsm. For experiments of high intracellular Cl^−^, KCl was increased to 78 mM while K-gluconate was reduced to 50 mM. Extracellular solution contained (mM): 140 NaCl, 4.7 KCl, 1.2 MgCl_2_, 2.5 CaCl_2,_ 10 HEPES and 10 glucose; pH was adjusted to 7.3 with NaOH and osmolarity was set to 315 mOsm. Extracellular solution was perfused at a continuous rate of 1–2 ml/min. To assess voltage gated Na^+^ currents in iPSC-sensory neurons, the internal solution contained (mM): 140 CsF, 10 NaCl, 1 EGTA and 10 HEPES. The extracellular solution contained (mM): 70 NaCl, 50 *N*-methyl-d-glucamine, 20 tetraethylammonium chloride, 1 CaCl_2_, 3 KCl, 1 MgCl_2_, 1 CaCl_2_, 10 HEPES, 10 glucose and 0.1 CdCl_2_. Ivermectin (Sigma-Aldrich) was diluted to a 10 000× stock in dimethyl sulphoxide (DMSO) fresh daily and added via the perfusion system. Unless otherwise stated, post-ivermectin recordings were always made 15 min after addition of the drug. In voltage clamp, ramps from −90 mV to −40 mV were used to measure membrane conductance. Ramps of 100 ms duration were delivered in 10 s intervals and conductance was derived from the linear portion of the resultant current slope. To assess voltage-gated Na^+^ currents potential was stepped to −85 mV for 8 s before a 20 ms test step to 0 mV, from a holding potential of −100 mV. Resting membrane potential was assessed in bridge mode, while firing properties were assessed in current clamp mode. Input resistance (R_Input_) was derived from the membrane potential deflection caused by a 20 pA hyperpolarizing current pulse at −60 mV. To determine rheobase, cells were depolarized from a holding potential of −60 mV by depolarizing current steps (50 ms) of increasing magnitude (Δ25 pA) until an action potential was generated. Spontaneous activity was defined as the presence of action potential firing during a 1 min bridge-mode recording sweep made immediately upon establishing whole cell access. Response to 10 s application of 1 μM capsaicin was assessed in bridge mode while recording from small diameter (<25 μM) neurons.

### Ca^2+^ imaging

For ratiometric Ca^2+^ imaging, cells were incubated with media containing 2 µM Fura-2 AM and 80 µM pluronic acid (ThermoFisher Scientific) for 1 h at 37°C, before being washed with extracellular fluid (in mM, 145 NaCl, 5 KCl, 10 HEPES, 10 d-glucose, 2 CaCl_2_ and 1 MgCl_2_, pH 7.4). For somal imaging, drug (vehicle or ivermectin) was added during the Fura-2 AM and pluronic acid incubation step. A 10× objective, dichroic LP 409 mirror and BP 510/90 emitter filter were used for Ca^2+^ imaging. Pairs of images using excitation filters BP 340/30 and BP 387/15, respectively, were captured every 2 s. Ratiometric 340/380 calculation was performed with a background subtraction. GluCl^+^ DRG were identified using BP 480/30 excitation filter, dichroic LP 505 mirror and BP 535/40 emission filter and labelled as such prior to recording protocols. Extracellular fluid was perfused with the addition of drugs by a gravity-driven application system with remotely controlled pinch valves. Therefore, the entire stimulation protocol and ivermectin application was performed in a time-locked and hands-free manner. Cells exhibiting a time-locked Ca^2+^ transient >0.2 ΔF/F were defined as having responded.

Compartmentalized microfluidic chambers were composed of a polydimethylsiloxane (PDMS) piece in which a pattern of channels and microgrooves were moulded that was bonded to a glass bottom culture dish. The fabrication of the master template in which the PDMS pieces were moulded from was performed as previously described in detail (Park *et al.*, 2006). Embryonic Day 16 rat DRG were processed for culture similarly to adult mouse tissue but with a reduced enzymatic digestion step of 45 min. We used rat DRG for this experiment as in our hands they grow through the microgrooves better and more consistently generate somal responses to terminal stimulation. Once dissociated embryonic rat DRG were resuspended in complete Neurobasal® medium and 10 000 cells were plated in a 4 µl volume in only one compartment using capillary action to draw the cell suspension along the entire compartment length. Ten microlitres of complete Neurobasal® medium was placed in the adjacent compartment. Cells were left to adhere for a minimum of 2 h before flooding the channels with complete Neurobasal® medium. To encourage axonal growth through the microgrooves we exploited the fluidic resistance to create a unidirectional NGF/GDNF gradient across the array. This was established according to Table 1.

**Table 1:** Chemoattraction protocol

	Somal compartment	Neurite compartment
**Day 0**	50 ng/ml NGF, 10 ng/ml GDNF in 200 µl	50 ng/ml NGF, 10 ng/ml GDNF in 200 µl
**Day 1**	5 ng/ml NGF, 1 ng/ml GDNF in 160 µl	50 ng/ml NGF, 10 ng/ml GDNF in 200 µl
**Day 2**	0 ng/ml NGF, 0 ng/ml GDNF in 160 µl	50 ng/ml NGF, 10 ng/ml GDNF in 200 µl
**Days 3 and 5**	0 ng/ml NGF, 0 ng/ml GDNF in 160 µl	50 ng/ml NGF, 10 ng/ml GDNF in 200 µl

DRG neurons were treated with AAV (GluClβ-AAV with or without GluClα-AAV) 3 days post-plating at an MOI of 100. Imaging experiments were performed 7 days post-plating. To identify neurons that had grown a neurite through the microgroove array, 100 µl of 1,1’-dioctadecyl-3,3,3’,3’-tetramethylindocarbocyanine perchlorate (DiI, Invitrogen, 40 µg/ml in complete Neurobasal® medium) was applied to the axonal compartment 2 h prior to Ca^2+^ imaging, with 200 µl of medium in the somal compartment to maintain fluidic isolation. Neurons that were positive for DiI and GluCl subunits (GluClα and GluClβ, or GluClβ only) were identified and subsequently used to assess Ca^2+^ response. Averages from one litter were used to compute group means and measures of group variance. Statistical comparisons were always made on the basis of litter number.

### Statistical analysis

Data are expressed throughout as mean ± standard error of the mean (SEM). Student’s *t*-test was used to compare the mean of two samples and one-way ANOVA for multiple comparisons. Where data related to pre- and post-ivermectin, a paired *t*-test was performed. Proportion of cells in excitable states was assessed by chi-squared test and the presence of spontaneous activity by Fischer’s exact *t*-test. For behavioural experiments over time, repeated measures ANOVA was used followed by *post hoc* Bonferroni test. Normality was assessed by Shapiro–Wilk test and non-parametric tests were used when appropriate (Wilcoxon signed-rank and Kruskal–Wallis tests). Statistical testing was performed with Excel or GraphPad Prism. *P* < 0.05 was taken as our threshold for significance.

## Results

### Evoked firing of sensory neurons is silenced by GluCl activation

**Figure 1:**
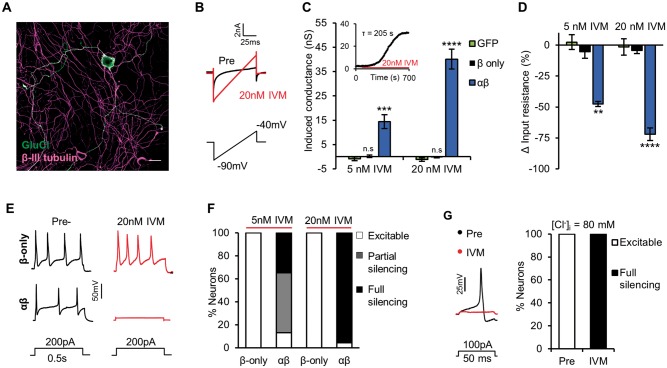
**GluCl activation silences electrical activity of DRG neurons *in vitro***. Whole-cell patch clamp recordings of DRG neurons electroporated with GluClα/β-YFP. (**A**) GluCl^+^ DRG neuron. Scale bar = 30 µm. (**B**) Voltage ramp used to determine membrane conductance. Ivermectin (IVM, 20 nM) treatment induces a membrane conductance in GluCl^+^ DRG, as seen by the increased slope of the current trace. (**C**) Induced conductance in response to ivermectin. (*Inset*) Example recording of ivermectin-conductance over time. One-way ANOVA with *post hoc* Bonferroni test comparing groups to control (GFP), GFP 5 nM ivermectin *n* = 10, GFP 20 nM ivermectin *n* = 10, β-only 5 nM ivermectin *n* = 11, β-only 20 nM ivermectin *n* = 15, αβ 5 nM ivermectin *n* = 24 and αβ 20 nM ivermectin *n* = 24. (**D**) Change in input resistance following ivermectin. One-way ANOVA with *post hoc* Bonferroni test comparing groups to control (GFP), GFP 5 nM ivermectin *n* = 10, GFP 20 nM ivermectin *n* = 10, β-only 5 nM ivermectin *n* = 11, β-only 20 nM ivermectin *n* = 15, αβ 5 nM ivermectin *n* = 24 and αβ 20 nM ivermectin *n* = 24. (**E**) Firing of neurons in response to supra-threshold current injection pre- and post-ivermectin treatment. (**F**) Ability of GluCl^+^ neurons to fire an action potential in response to a short (50 ms) depolarizing current. Neuronal excitability was defined by the fold-increase in rheobase comparing pre- and post-ivermectin values: <3 (excitable), 3–10 (partial silencing) or >10-fold (full silencing). *P* < 0.001, chi-squared test (*χ*^2^ = 55.74 with 4 degrees of freedom). β-only 5 nM ivermectin *n* = 11, β-only 20 nM ivermectin *n* = 15, αβ 5 nM ivermectin *n* = 24 and αβ 20 nM ivermectin *n* = 24. (**G**) Excitability of GluCl^+^ DRG assessed while using an intracellular solution containing 80 mM Cl^−^. (*Left*) Example firing of a GluCl^+^ neuron in response to supra-threshold current stimuli pre- and post-ivermectin (20 nM). (*Right*) Following ivermectin, 5/5 neurons were unable to fire an action potential in response to a current 10-fold their pre-drug rheobase value (full silencing). All grouped data are mean ± SEM, *P < 0.05, **P < 0.01, ***P < 0.001; *P* < 0.0001.

To assess the efficacy of GluCl for silencing sensory neurons, we first performed whole-cell patch clamp recordings of dissociated mouse DRG neurons electroporated with GluCl plasmid DNA. At rest, neurons expressing both α- and β-subunits of GluCl (Fig. 1A) had similar membrane and firing properties to control (neurons electroporated with GFP-only vector) (Supplementary Table 1), suggestive of no GluCl activity. However, low levels of ivermectin induced a slow, stable and dose-dependent conductance (Fig. 1B and C). Neurons expressing only the β-subunit of GluCl did not respond to ivermectin, consistent with previous observations that this subunit does not form functional ivermectin-sensitive homomers (Frazier *et al.*, 2013). This validated our use of GluCl β-only as a suitable control in future experiments. Unlike CNS neurons, sensory neurons maintain a high intracellular Cl^−^ concentration ([Cl^−^]_i_) (Gilbert *et al.*, 2007) and as such, initiating a Cl^−^ conductance would not be expected to inhibit activity by the classical fashion of hyperpolarizing the membrane potential (V_m_). We reasoned, however, that the large drop in membrane resistance associated with GluCl activation (Fig. 1D) would be sufficient to inhibit neuronal activity. Indeed under our recording conditions ([Cl^−^]_i_ = 30 mM) GluCl activation potently reduced evoked firing. After 15 min treatment with 20 nM ivermectin, 95.83% (23/24) of GluCl^+^ neurons did not generate an action potential in response to a current stimulus 10-fold the pre-ivermectin rheobase value, while control neurons were unaffected (Fig. 1E and F). The transient opening of Cl^−^ channels can depolarize and activate sensory neurons (Cho *et al.*, 2012). However, we noted no action potential firing in the 15 min prelude to assessment of evoked firing in GluCl^+^ neurons (5 nM ivermectin *n* = 13 cells, 20 nM ivermectin *n* = 13). Cells recorded from ranged in diameter from 15 to 40 μm and silencing was effective in all sizes of neuron (Supplementary Fig. 1). DRG [Cl^−^]_i_ levels are heterogeneous (Gilbert *et al.*, 2007) and can be increased after nerve injury (Pieraut *et al.*, 2007), altering the polarity and drive of Cl^−^ flux. Under recording conditions of high intracellular Cl^−^ ([Cl^−^]_i_ = 80 mM, E_Cl^−^_ = −16 mV) ivermectin depolarized V_m_, but was still effective at silencing neuronal activity (Fig. 1G and Supplementary Fig. 2). These results indicate that even when providing a depolarizing drive, GluCl activity can silence evoked electrical activity of sensory neurons.

### GluCl silencing is effective at the axon as well as the soma

**Figure 2:**
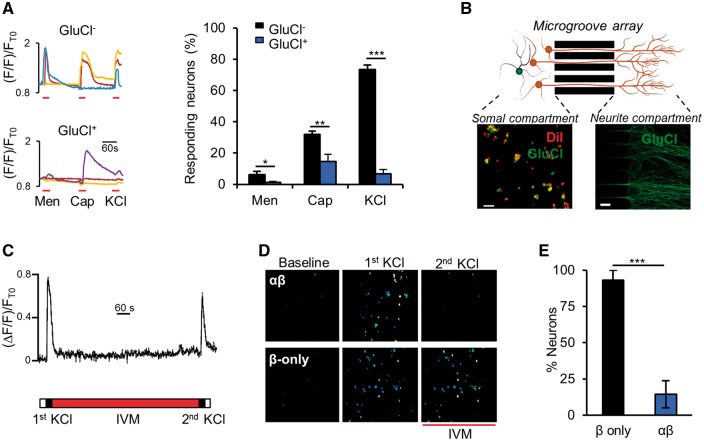
**GluCl mediates local silencing in response to excitatory stimuli.** (**A**, *left*) Representative somal Ca^2+^ responses of ivermectin-treated DRG neurons to consecutive stimulation with 200 μM menthol (Men), 1 μM capsaicin (Cap) and 50 mM KCl. (*Right*) Proportion of neurons that responded to each stimuli. One-way ANOVA with *post hoc* Bonferroni test. Data derived from *n* = 9 independent experiments (GluCl^+^, total of 162 neurons, GluCl^−^, 629 neurons). (**B**) Schematic of experimental set-up illustrating somal and neurite compartments separated by a microgroove array. DiI treatment of the neurite compartment marks GluCl^+^ cell bodies that have successfully sent axonal projections through the microgroove array. GluCl^+^ axons are clearly discernable in the neurite compartment. Scale bars = 50 µm. (**C**) Somal Ca^2+^ transients of a control neuron in response to the experimental stimulation protocol [two KCl (50 mM) neurite stimulations separated by 12 min ivermectin (20 nM) wash-on only to the neurite compartment]. This set-up allows us to assess the effect of local drug administration to axonal activation. (**D**) Ratiometric Ca^2+^ responses of DRG cell bodies while neurites are stimulated in isolated compartments, before and after acute ivermectin treatment of neurites. (**E**) Percentage of cell bodies that responded to the second neurite depolarization stimulus, as a proportion of those that responded to the first stimulation. Student’s unpaired *t*-test, *n* = 4 independent experiments (β-only, total of 138 cells, αβ, 200 cells). All grouped data are mean ± SEM, *P < 0.05, **P < 0.01, ***P < 0.001.

To circumvent confounds of whole-cell patching and artificial modulation of intracellular ionic concentrations; we performed Ca^2+^-imaging experiments. The proportion of GluCl^+^ neurons responding to KCl-induced depolarization and naturally occurring TRP channel agonists [menthol (McKemy *et al.*, 2002) and capsaicin (Caterina *et al.*, 1997)] following ivermectin was markedly reduced compared to control, indicative of effective silencing (Fig. 2A). The fact that the Ca^2+^ response to capsaicin was not completely abolished likely represents the direct entry of Ca^2+^ through TRPV1 (a non-selective cation channel). This notion is supported by our finding that GluCl^+^ neurons did not fire action potentials in response to capsaicin, following 20 nM ivermectin (Supplementary Fig. 3). Whether GluCl activation silences neurons solely at the level of the soma or also blocks axonal action potential propagation has not previously been determined; but the question is highly relevant to sensory neurons given their long axonal projections. We used microfluidic compartments to separate the soma and neurites of GluCl^+^ neurons (Fig. 2B) (Park *et al.*, 2006). Local depolarization of neurites leads to Ca^2+^ transients in DRG cell bodies dependent on axonal transmission (Fig. 2C). Ivermectin applied only to neurite endings was able to almost entirely block soma responses to neurite depolarization (Fig. 2D and E), suggesting that GluCl activation can interfere with primary afferent transmission at the level of the axon and not just the soma. The *in vitro* data illustrate that GluCl activation can potently silence sensory neurons and compelled us to assess whether the system was applicable to the *in vivo* setting.

### Intrathecal AAV effectively delivers GluCl to sensory neurons *in vivo*

**Figure 3:**
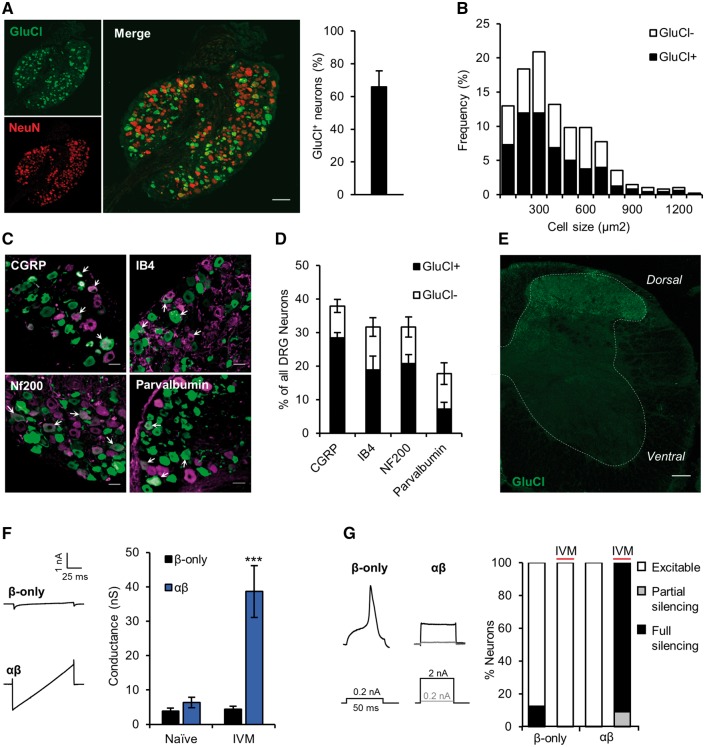
**Intrathecal delivery of GluCl-AAV efficiently transduces sensory neurons.** Analysis of tissue taken from animals 4 weeks following intrathecal co-delivery of AAV9-GluClα and AAV9-GluClβ. (**A**, *left*) L4 DRG immunostained for YFP (green) and NeuN (red). Scale bar = 100 μm. (*Right*) GluCl^+^ DRG as a proportion of total neurons. Data represent mean ± SEM from three animals and a total of 912 neurons. (**B**) Size distribution of GluCl^+^ neurons. Data pooled from three animals and represent a total of 379 cells. (**C**) GluCl (green) co-localization with DRG subpopulation markers (magenta). White arrows show double-labelled neurons. Scale bar = 30 µm. (**D**) Percentage of GluCl^+^ DRG in the different subpopulations. Data represent results from *n* = 3 animals and a total of >600 cells/group. (**E**) Representative lumbar spinal cord immunostained for YFP (*n* = 4 animals assessed). Scale bar = 100 µm. (**F**) Membrane conductance of *ex vivo* GluCl^+^ neurons taken from GluCl-AAV animals untreated (Naïve) and 24 h following 5 mg/kg i.p. ivermectin (IVM). (*Left*) Current traces in response to membrane voltage ramp −90 to −40 mV (0.5 mV/ms) of neurons from ivermectin treated animals. (*Right*) Quantification of membrane conductance. One-way ANOVA followed by *post hoc* Bonferroni test comparing groups to control (Naive β-only), naive β-only, *n* = 8; naïve αβ, *n* = 7; ivermectin β-only, *n* = 8; ivermectin αβ, *n* = 10. (**G**) Firing properties of GluCl-AAV animal derived GluCl^+^ neurons. (*Left*) Action potential traces in response to depolarizing current injection of DRG neurons derived from ivermectin-treated animals. (*Right*) Excitability status as defined by a rheobase value: <3 (excitable), 3–10 (partial silencing) or >10 (full silencing) standard deviations above the mean of control (Naïve β-only, mean = 160.1 ± 160.6 pA). *P* < 0.001, chi-squared test (*χ*^2^ = 32.52 with 6 degrees of freedom); Naive β-only, *n* = 8; Naïve αβ, *n* = 7; ivermectin β-only, *n = *8; ivermectin αβ, *n* = 10. All averaged data represent the mean ± SEM. ***P < 0.001.

Following nerve injury, evidence exists that both myelinated (Wall and Gutnick, 1974; Wall and Devor, 1983; Kajander and Bennett, 1992) and unmyelinated afferents (Wu *et al.*, 2001; Serra *et al.*, 2012) are involved in the generation of neuropathic pain (Ossipov *et al.*, 1999; Tarpley *et al.*, 2004; Xu *et al.*, 2015). We therefore aimed to deliver GluCl to a broad range of sensory neurons *in vivo*. We engineered AAV9 to express GluClα and GluClβ separately; under the control of the ubiquitous CAG promoter and modified GluClα to contain a monomeric cerulean fluorophore in the place of YFP to better visualize subunit co-localization (Supplementary Fig. 4). Fluorophore switch had no effect on channel function (Supplementary Fig. 4). We used intrathecal delivery to selectively transduce neurons of the sensory ganglia while avoiding transduction of spinal cord neurons (Mason *et al.*, 2010; Jacques *et al.*, 2012). Four weeks post-surgery, GluCl was expressed in 66.1 ± 9.6% of L4 DRG neurons (*n* = 4 animals with one excluded from analysis due to zero expression) (Fig. 3A). Size frequency analysis of GluCl^+^ neurons revealed equally distributed transduction of neurons across all diameters (Fig. 3B). Consistent with this result, co-labelling experiments showed that transduction rates were similar across different molecularly defined sub-populations (Fig. 3C and D) and was not selective for a particular population. Homomeric GluCl channels provide little or no conductance in response to ivermectin (Frazier *et al.*, 2013); however, we found that the vast majority (78.9%) of GluCl^+^ neurons expressed both subunits (Supplementary Fig. 5). GluCl^+^ afferent endings were seen in superficial and deep dorsal horn laminae but crucially no expression was seen in ventral horn motor neurons (Fig. 3E), brain or autonomic structures (Supplementary Fig. 6). In the periphery we observed GluCl expression in neurite endings of the skin and deep dermal nerve fibres (Supplementary Fig. 7). Channel activity remains for several days post-ivermectin dosing due to slow dissociation from the channel (Slimko *et al.*, 2002) and tissue retention of ivermectin (Lerchner *et al.*, 2007). This allowed us to test silencing efficacy following AAV delivery and agonist treatment *in vivo*. GluCl-AAV animals were dosed with 5 mg/kg ivermectin i.p. 24 h prior to dissociation of lumbar DRG. *Ex vivo* GluCl^+^ DRG neurons exhibited analogous features to those observed *in vitro*; a substantive basal conductance and an inability to generate action potentials in response to current injection (Fig. 3F and G). Neurons did not exhibit a significantly different resting membrane potential (β-only naïve, −59.39 ± 2.23 mV versus αβ ivermectin, −56.01 ± 1.9, *P* = 0.18 Student’s *t*-test.) AAV is considered a suitable vehicle to achieve long-term expression of exogenous transgenes in target tissue (Hadaczek *et al.*, 2010), which makes it a highly attractive approach from a translational perspective. However, there are previous reports of partial shutdown of transgene expression over time when AAV is used to target DRG (Mason *et al.*, 2010; Iyer *et al.*, 2014). To assess expression longevity, we maintained a small cohort of GluCl-AAV animals for 7 months and found robust expression still evident (Supplementary Fig. 8). We noted no reduction in cutaneous afferent innervation or upregulation of the injury marker ATF3 (Supplementary Fig. 9).

### *In vivo* silencing elevates mechanical and thermal pain thresholds

**Figure 4:**
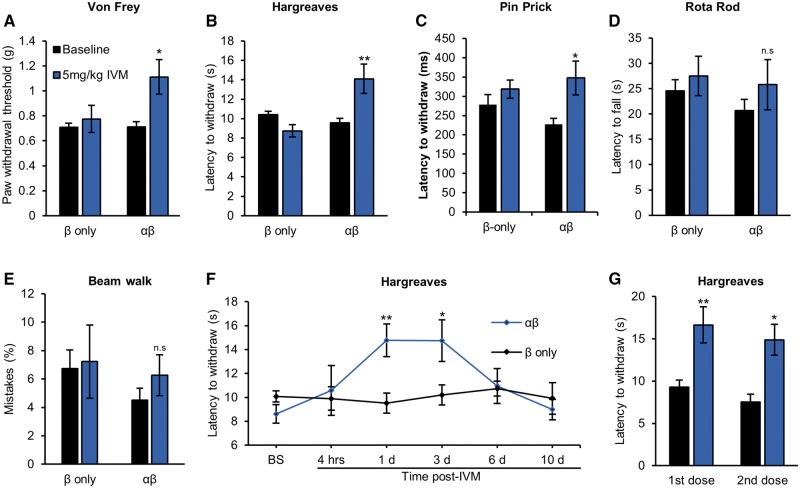
**GluCl activation increases thermal and mechanical pain thresholds.** (**A**) Von Frey test of mechanical sensitivity (β only, *n* = 14 animals and αβ, *n* = 13); (**B**) Hargreaves test of thermal sensitivity (β only, *n* = 14 animals and αβ, *n* = 13); (**C**) Pinprick assay of noxious mechanosensation (β only, *n* = 11 animals and αβ, *n* = 10); (**D**) Rotarod test of motor performance (β only, *n* = 14 animals and αβ, *n* = 13); and (**E**) beam walk test of proprioception (β only, *n* = 8 animals and αβ, *n* = 9) of GluCl AAV animals before and 24 h following 5 mg/kg ivermectin (IVM). Student’s paired *t*-test. (**F**) Time course of thermal sensitivity following a single dose of systemic ivermectin. Repeated measures ANOVA followed by *post hoc* Bonferroni test comparing β only (*n* = 12) and αβ (*n* = 10.) (**E**) Ivermectin-induced change in thermal sensitivity of two separate doses of ivermectin given 24 h prior to testing, performed 3 weeks apart. Student’s paired *t*-test, *n* = 6 animals. All averaged data represent mean ± SEM. *P < 0.05, **P < 0.01. BS = baseline.

To determine whether GluCl activation was sufficient to alter sensory thresholds, we performed tests of reflex withdrawal to mechanical (von Frey), thermal (Hargreaves) and noxious mechanical (pinprick) stimuli. Twenty-four hours post-ivermectin treatment mechanical thresholds of GluCl^+^ animals were increased by 63.04 ± 20.86% (*P* = 0.015) (Fig. 4A), thermal thresholds were increased by 48.4 ± 15.62% (*P* = 0.007) (Fig. 4B) and latency to withdraw from a noxious mechanical stimulus by 62.2 ± 8.3% (*P* = 0.034) (Fig. 4C). Motor performance and proprioception (as assessed by the rotarod and beam walk tests, respectively) were not affected in either experimental or control groups following ivermectin (Fig. 4D and E). We investigated in detail the time course of GluCl-mediated behavioural responses. After a single dose, hypo-responsiveness was apparent by 24 h and was maintained for 3 days before thresholds returned to baseline by 6 days (Fig. 4F). Once GluCl channels have closed following ivermectin washout they should be amenable to reactivation by a subsequent dose of the drug (Lerchner *et al.*, 2007). Indeed, we found that a second ivermectin administration had similar efficacy to the first after an interval of 3 weeks (Fig. 4G). These results highlight GluCl silencing of DRG neurons as a means to non-invasively manipulate sensory thresholds in a chronic and reproducible fashion.

### GluCl activation reverses traumatic nerve injury-induced hypersensitivity

**Figure 5:**
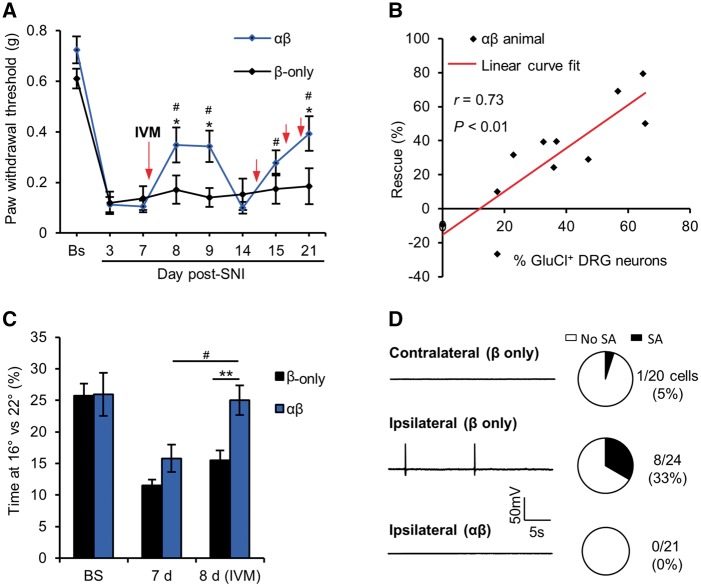
**GluCl activation can reverse nerve injury-induced hypersensitivity.** (**A**) Time course of mechanical hypersensitivity following SNI. Red arrows indicate the time at which animals received a systemic dose of 5 mg/kg ivermectin. *P < 0.05, repeated measures (RM)-ANOVA followed by *post hoc* Bonferroni test comparing means of β-only and αβ groups. ^#^*P* < 0.05, repeated measures ANOVA followed by *post hoc* Bonferroni test comparing post-SNI time-points with Day 7, *n* = 11 animals per group. (**B**) Correlation between average rescue (Days 8 and 15) of mechanical hypersensitivity following a single dose of ivermectin and the proportion of L4 DRG neurons expressing GluCl. *P < 0.05, Pearson’s correlation co-efficient. (**C**) Time spent at 16°C versus 22°C drops 7 days after SNI but is recovered 24 h following systemic ivermectin. **P < 0.01, *P < 0.05, repeated measures ANOVA followed by *post hoc* Bonferroni test comparing means of β-only and αβ groups. ^#^*P* < 0.05, repeated measures ANOVA followed by *post hoc* Bonferroni test comparing means at Day 7 versus Day 8, *n* = 11 animals per group. (**D**, *left*) traces of GluCl^+^ neurons taken from uninjured (contralateral) and injured (ipsilateral) DRG of SNI animals illustrating resting membrane potential over 30 s. (*Right*) Proportion of cells exhibiting ectopic activity. **P < 0.01, chi-squared test (*χ*^2^ = 12.01 with 2 degrees of freedom), data derived from three independent experiments. All averaged data represent mean ± SEM. SA = spontaneous activity.

Chronic pain following nerve injury is driven by hyperexcitability (Smith *et al.*, 2013) and ectopic activity of primary afferents (Haroutounian *et al.*, 2014; Vaso *et al.*, 2014). We asked whether our approach of long-term electrical silencing of afferents would be sufficient to counter this phenomenon in animals that had undergone SNI (Decosterd and Woolf, 2000). We found that GluCl activation could repetitively reverse established mechanical hypersensitivity following SNI (Fig. 5A and Supplementary Fig. 10). *Post hoc* tissue analysis revealed that the degree of rescue correlated well with the proportion of L4 DRG cells expressing GluCl (Fig. 5B), supporting the view that primary afferent drive is critical for the maintenance of the neuropathic pain state. We used a two-chamber cold place preference as an operant measure of thermal hypersensitivity following nerve injury (Balayssac *et al.*, 2014). One week after SNI, animals spend significantly less time in the cold (16°C chamber versus 22°C) than pre-injury levels. Ivermectin reversed the observed cold allodynia in the experimental group but had no effect on controls (Fig. 5C). As a cellular correlate, we studied DRG neurons taken from chronic SNI animals (25–33 days post-SNI) for their electrophysiological properties *in vitro*. *Ex vivo* neurons derived from injured DRG had a higher incidence of ectopic activity than control [ipsilateral (8/24 cells) versus contralateral (1/20 cells), *P* = 0.027 Fischer’s exact *t*-test]. However, injured neurons from ivermectin-treated GluCl^+^ animals exhibited no ectopic activity (Fig. 5D) and largely silenced evoked activity (Table 2), indicative of robust silencing. GluCl activation is therefore sufficient to silence nerve-injury induced ectopic activity of primary afferents and reverse pain related behaviour.

**Table 2:** Biophysical and firing properties of DRG neurons from SNI animals

	β only			αβ	
	Contralateral	Ipsilateral	*P*-value	Ipsilateral	*P*-value
**Resting membrane potential (mV)**	−57.15 ± 1.46	−56.68 ± 1.79	>0.99	−51.17 ± 2.24	0.08
**Input resistance (MΩ)**	193.33 ± 20.44	183.33 ± 22.76	>0.99	75.97 ± 7.95***	<0.001
**Rheobase (pA)**	146.31 ± 15.15	181.21 ± 32.69	>0.99	1476.22 ± 313.79***	<0.001
**Capacitance (pF)**	31.49 ± 1.84	32.53 ± 2.30	>0.99	31.3 ± 2.76	>0.99
**Number of cells**	20	24		21	

***P < 0.001, one-way ANOVA comparing group versus control (β-only contralateral), followed by *post hoc* Bonferroni test. Only αβ neurons that fired an action potential contributed to rheobase values (12/21 cells).

### GluCl silences human iPSC-derived sensory neurons

**Figure 6:**
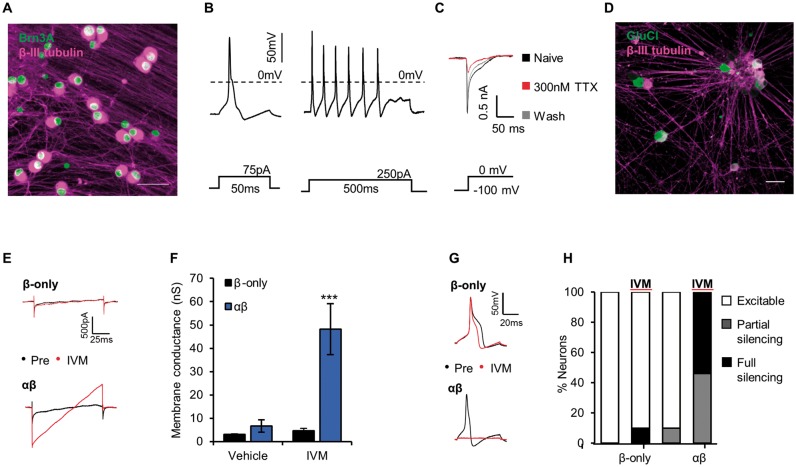
**GluCl activation silences human iPSC-derived sensory neurons.** (**A**) Mature human iPSC-derived sensory neurons stain positive for the sensory neuron transcription factor Brn3a (green) and neuronal marker β-III tubulin (magenta). Scale bar = 50 μm. (**B**) Evoked firing in response to short (*left*) and long (*right*) depolarizing current injection. Note the inflection on the falling phase of the action potential—a property of endogenous nociceptors. (**C**) Example voltage clamp recording demonstrating both a TTX-sensitive and a TTX-resistant component to the Na^+^ current evoked by membrane depolarization to 0 mV. (**D**) GluCl expression in human iPSC-sensory neurons 2 weeks following treatment with GluCl-AAV. Scale bar = 50 μm. (**E**) Voltage ramp recordings of neurons expressing GluCl and treated with ivermectin (IVM, 20 nM). (**F**) Quantification of membrane conductance of neurons pretreated with either ivermectin (20 nM) or vehicle (0.01% DMSO). Kruskal-Wallis followed by *post hoc* Dunn’s test comparing groups to control (vehicle treated β-only): vehicle β-only, *n* = 9; vehicle αβ, *n* = 11; ivermectin β-only, *n* = 11; and ivermectin αβ, *n* = 13. (**G**) Evoked firing in response to supra-threshold current injection pre- and post-ivermectin treatment. (**H**) Neuronal excitability as defined based on a rheobase value; <3 (excitable), 3–10 (partial silencing) or >10 (full silencing) standard deviations above the mean of control (vehicle treated β-only, mean = 103 ± 28.2 pA). *P* < 0.001, chi-squared test (*χ*^2^ = 32.45 with 6 degrees of freedom); vehicle β-only, *n* = 9; vehicle αβ, *n* = 11; ivermectin β-only, *n* = 11; and ivermectin αβ *n* = 13. All grouped data are mean ± SEM, ***P < 0.001.

The use of human model systems can potentially improve translation of preclinical findings in animal models into clinical therapeutics (Woolf, 2010; Percie Du Sert and Rice, 2014). To address whether the GluCl system may have efficacy in the context of human sensory neurons, we generated cultures of human iPSC-derived sensory neurons. We followed a differentiation protocol known to produce neurons with an expression profile and functional characteristics that are very similar to rodent sensory neurons (Chambers *et al.*, 2012; Young *et al.*, 2014; Clark *et al.*, 2017). Resultant neurons uniformly express the sensory neuron marker Brn3a, project extensively arborized neurites and exhibit mature electrophysiological characteristics (Fig. 6A–C). Analogous to mouse DRG, human iPSC-sensory neurons expressing GluCl and treated with ivermectin show a substantial conductance (Fig. 6D–F) and limited capacity to generate action potentials to otherwise suprathreshold current stimuli (Fig. 6G and H). These results indicate the generality of the GluCl system and its potential relevance for use in human sensory neurons.

## Discussion

To summarize, we show that activation of GluCl channels results in robust silencing of rodent and human iPSC-derived sensory neurons. AAV-mediated delivery is capable of highly specific, efficient and long-lasting expression of GluCl in sensory neurons *in vivo* without deficits in cutaneous afferent innervation or upregulation of the injury marker ATF3 and can normalize pain-related behaviour following peripheral nerve injury in a reproducible and reversible fashion.

The GluCl channel that we used in this study is a heteromeric glutamate-gated chloride channel modified so be activated by low doses of ivermectin, but is insensitive to its natural ligand glutamate (Slimko *et al.*, 2002). The initial version (GluCl v1.0) has previously been used to silence neurons in the CNS (Lerchner *et al.*, 2007; Lin *et al.*, 2011). In this study we used a recently modified version (GluCl v2.0), which shows higher sensitivity to ivermectin and improved trafficking of the β-subunit (Frazier *et al.*, 2013), but has (up until now) not been tested *in vivo* or in the peripheral nervous system. We were struck by the efficacy of GluCl v2.0 in silencing rodent sensory neurons both *in vitro* and *ex vivo.* In the absence of ligand, neurons expressing GluCl were electrically normal, but treatment with low levels of ivermectin evoked a substantive membrane conductance that rendered almost all neurons entirely resistant to evoked firing.

Systems designed to inhibit neuronal activity are classically designed to hyperpolarize the membrane potential (Lechner *et al.*, 2002; Slimko *et al.*, 2002; Armbruster *et al.*, 2007) and render the cell less likely to reach firing threshold. In CNS neurons, Cl^−^ has a hyperpolarized Nernst potential due to low [Cl^−^]_i_ levels and therefore an increased conductance will hyperpolarize the resting membrane potential (Lerchner *et al.*, 2007). DRG neurons are unique in that they actively maintain higher [Cl^−^]_i_ through activity of several Cl^−^ co-transporters (Sung *et al.*, 2000). Consistent with this we found that GluCl activation did not hyperpolarize the membrane potential; however, we still noted substantial silencing efficacy. This may be explained by the marked reduction in membrane resistance associated with GluCl activation, resembling the phenomena of shunting inhibition observed in endogenous neural systems (Segev, 1990; Borg-Graham *et al.*, 1998). Despite this, the precise mechanism of silencing would prove challenging to demonstrate empirically.

Ca^2+^ influx to the soma in response to natural stimuli [menthol and capsaicin, which activate subsets of small diameter DRG cells (Caterina *et al.*, 1997; McKemy *et al.*, 2002)] was reduced in GluCl-activated neurons. Given the long pseudo-unipolar axons of sensory neurons, there are advantages to a silencing system that can act on neurites as well as at the level of the soma. Using a microfluidic platform in which soma and neurites can be isolated and treated independently, we show that ivermectin application to sensory neurites alone is sufficient to prevent action potential propagation to the soma following peripheral depolarization.

With a view to translational applications and to test whether there were cross-species differences in silencing efficacy, we investigated whether the GluCl system could silence human iPSC-derived sensory neurons. We used a previously published differentiation protocol known to generate sensory neurons (Chambers *et al.*, 2012) that has been shown to be a valuable model of human pain disorders (Young *et al.*, 2014; Cao *et al.*, 2016). Ivermectin treatment of human iPSC-derived sensory neurons expressing GluCl evokes a membrane conductance and silences these neurons to a similar extent as their rodent analogues.

Ivermectin does not readily cross the blood–brain barrier (Schinkel *et al.*, 1994), leading previous studies of the GluCl system in the CNS to use relatively high dosing regimes (Lerchner *et al.*, 2007). When administering ivermectin *in vivo* we opted to use the lower end of the dose range (5 mg/kg) as our target was peripheral neurons. This lower dose reduces the likelihood of off-target effects given that at high doses ivermectin can bind to GABA_A_ receptors (Dawson *et al.*, 2000). Doses of ivermectin <10 mg/kg have been shown not to have CNS depressant effects (Trailovic and Nedeljkovic, 2011). Reassuringly we did not observe any effects of ivermectin at a dose of 5 mg/kg on sensory or motor function in control animals (either in the naïve state or following nerve injury). In experimental animals expressing GluCl, a single dose of ivermectin resulted in a significant elevation of reflex withdrawal thresholds to noxious mechanical and thermal stimuli lasting 3 days. This is similar to the long-lasting behavioural effects previously observed following use of the GluCl v1.0 system in the striatum (Lerchner *et al.*, 2007) and is thought to possibly reflect slow dissociation of ivermectin from GluCl (Slimko *et al.*, 2002) and/or long-lasting retention of ivermectin in tissue (Lerchner *et al.*, 2007). Importantly we noted that not only were the effects reversible but could also be reproduced following a subsequent treatment. We did not observe any motor deficits.

Finally, we tested the efficacy of GluCl in the spared nerve injury model of neuropathic pain (Decosterd and Woolf, 2000). Importantly, we treated with ivermectin at 1 week post-injury, a time when mechanical and cold hypersensitivity is well established. Ivermectin could significantly ameliorate mechanical pain-related hypersensitivity as well as reversing cold allodynia. This effect was reversible and reproducible up to 3 weeks post-injury, which was the last time point of testing. There was a significant correlation between the proportion of DRG cells transduced with GluCl and the degree of reversal of mechanical pain-related hypersensitivity. Consistent with these behavioural findings, we found that when assessed *ex vivo* ivermectin had successfully suppressed both ectopic and evoked activity in those sensory neurons expressing GluCl. Given the selectivity of AAV delivery for sensory neurons these findings emphasize the importance of aberrant primary afferent input in the maintenance of neuropathic pain (Vaso *et al.*, 2014). The past decade has seen exciting developments in the identification of ion channels/signalling pathways contributing to the development and maintenance of neuropathic pain (Waxman and Zamponi, 2014; Habib *et al.*, 2015). A caveat to therapeutically targeting individual molecules is the proposed degeneracy in the development of neuronal hyperexcitability following nerve injury (Ratte *et al.*, 2014). GluCl activation represents a strategy to circumvent this problem by silencing neuronal activity regardless of inherent dysregulation in the system. This property may make the system also appropriate for other neurological disorders whereby dampening of neuronal excitability could prove efficacious, such as epilepsy (Avaliani *et al.*, 2016).

This is the first demonstration of efficacy of a chemogenetic system in a chronic pain model. There is some evidence that expression of the designer inhibitory G-protein-coupled receptor hM4Di (inhibitory designer receptor exclusively activated by designer drug DREADD) (Armbruster *et al.*, 2007) in primary afferents can alter acute pain thresholds *in vivo* (Iyer *et al.*, 2016; Saloman *et al.*, 2016), however although widely used in the CNS its applicability to peripheral sensory neurons is questionable. Mouse sensory neurons do not exhibit functional GIRK currents (Nockemann *et al.*, 2013), thought to be the endogenous mediator of hM4Di silencing (Armbruster *et al.*, 2007). This contrasts with rat and human sensory neurons wherein at least a proportion of sensory neurons do express GIRK channels (Nockemann *et al.*, 2013). In recordings from mouse DRG cells expressing hM4Di the reported efficacy was limited with <50% of such neurons showing a change in excitability (Saloman *et al.*, 2016). These authors have also shown that hM4Di expression in primary afferents alters Na^+^ and Ca^2+^ currents in the absence of the ligand (Clozapine-N-oxide, CNO) and inhibits the analgesic effect of endogenous inhibitory G-protein-coupled receptors (a major confound if this were ever to be used therapeutically). Finally there are also concerns about the safety of the receptor ligand, CNO (Jann *et al.*, 1994; Chang *et al.*, 1998; Maclaren *et al.*, 2016).

Other forms of neuromodulation include electrical stimulation devices (Colloca *et al.*, 2017) and optogenetics (which has not yet been applied to patients). Optogenetics offers exciting opportunities but also faces many challenges, largely based on safely delivering sufficient illumination to target tissues (Montgomery *et al.*, 2016). The chemogenetic approach has potential advantages, for instance in the ability to silence deep afferents inaccessible to light, which are a key source of ectopic activity (Michaelis *et al.*, 2000; Kirillova *et al.*, 2011), without the need of implants. One disadvantage is that ivermectin-mediated silencing *in vivo* exhibits temporal imprecision; however, the fact that behavioural efficacy is maintained for 3 days following a single dose could be advantageous when treating disorders of persistent hyperexcitability such as neuropathic pain. Neuromodulation mediated by electrical stimulation is used in the treatment of neuropathic pain (Kumar and Rizvi, 2014). However, electrode insertion is invasive, positive sensory symptoms may be troubling and efficacy is limited.

There would be significant challenges in developing a clinical therapeutic using our approach, mainly relating to delivery of the GluCl channel to sensory neurons in patients. Implicit in the findings from our nerve injury experiment is the fact that GluCl efficacy is directly correlated with the level of transduction achieved. AAV is, however, well tolerated in humans, can transfect post-mitotic neurons with long lasting expression (Hadaczek *et al.*, 2010) and is already used in clinical trials (Kotterman and Schaffer, 2014). GluCl is modified from a *Caenorhabiditis elegans* protein and so the risk of an immune response in humans would need to be carefully considered. This is a major challenge for the field of AAV gene therapy, but is particularly pertinent when expressing a non-self transgene in the periphery (Montgomery *et al.*, 2016). It is encouraging that long-term GluCl expression does not cause overt injury to DRG neurons and most significantly does not result in cutaneous denervation in our mouse models. However, strategies to modulate any potential immune response [e.g. tolerance induction or immune suppression (Mingozzi *et al.*, 2003; Sack and Herzog, 2009)] to GluCl would have to be considered as part of any translational pathway. There is evidence that ectopic activity develops in both small and large diameter sensory afferents and that both populations contribute to neuropathic pain (Ossipov *et al.*, 1999; Tarpley *et al.*, 2004; Xu *et al.*, 2015), hence our logic in targeting all sensory neurons. In certain situations, such as inflammatory pain (Abrahamsen *et al.*, 2008; Brenneis *et al.*, 2013), there would be an argument to restrict GluCl expression to small diameter nociceptive afferents that would necessitate development of effective promoters restricted to this population. The need to generate two independent AAVs expressing each subunit is a disadvantage when trying to silence a broad range of sensory neurons; however, certain paradigms can exploit this by using two independent promoters to express the subunits in an intersectional manner (Haubensak *et al.*, 2010). Our data provide proof-of-concept that this chemogenetic approach can be used to define primary afferent contributions to persistent pain states and furthermore has translational potential to suppress hyperexcitability and treat neuropathic pain.

## Supplementary Material

Supplementary Tables and Figures

## Abbreviations

AAV: adeno-associated virus
DRG: dorsal root ganglia
GluCl: glutamate-gated chloride channel
iPSC: induced pluripotent stem cell
SNI: spared nerve injury
