# Supplementary material for: Using an engineered glutamate-gated chloride channel to silence sensory neurons and treat neuropathic pain at the source
Source: Brain. 2017 Aug 19;140(10):2570–85. doi: 10.1093/brain/awx201 (PMC5841150; doi:10.1093/brain/awx201)
Supplement: Supplementary Tables and Figures [file awx201_supp_tables_figures.pdf]

Using an engineered GluCl channel to silence sensory neurons and treat neuropathic pain at the source

G. A. Weir[1], S. J. Middleton[1]†, A. J. Clark[1]†, T. Daniel[1], N. Khovanov[2], S. B. McMahon[2] and D. L. Bennett[1]*

Supplemental information

	GFP	β-only	αβ
**Membrane conductance (nS)**	6.25 ± 1.04	5.81 ± 0.87	5.39 ± 0.49
**Resting membrane potential (mV)**	-60.25 ± 1.62	-60.6 ± 1.91	-60.15 ± 1.43
**Rheobase (pA)**	403.54 ± 105.76	330.32 ± 59.96	387.21 ± 73.16
**Capacitance (pF)**	35.15 ± 3.74	34.71 ± 3.68	33.29 ± 4.68
**Number of cells**	20	26	48

Supplementary Table 1. Biophysical and evoked firing properties of untreated DRG neurons expressing GFP, GluCl*β* or GluCl*αβ*. No statistical difference was observed for any measured parameter**.** One-way ANOVA comparing group vs control (GFP), followed by post-hoc Bonferroni test

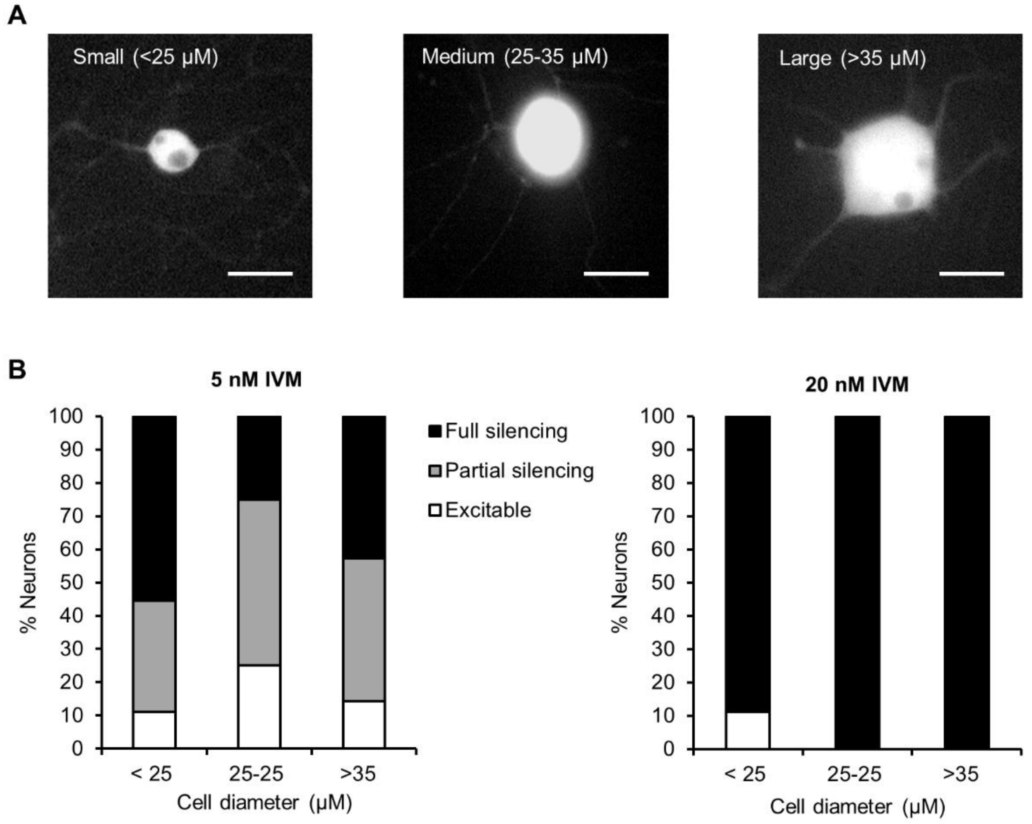

Supplementary Figure 1. GluCl silencing is effective in DRG of all sizes. (**A**) Representative fluorescent images of small, medium and large diameter $GluCl^{+}$ DRG neurons. Scale bar represents 25 µm. (**B**) Silencing efficacy of GluCl activation stratified by cell size with data derived from Fig 1F. Neuronal excitability was defined by the fold-increase in rheobase comparing pre- and post-IVM values: <3 (*excitable*), 3-10 (*partial silencing*) or >10-fold (*full silencing*). 5 nM IVM, *n* = 9 small, 8 medium and 7 large cells. 20 nM IVM**,** *n* = 9 small, 8 medium and 6 large cells.

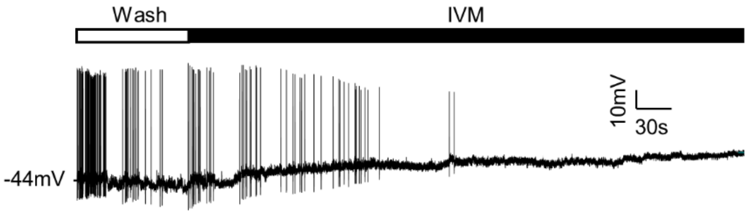

Supplementary Figure 2. GluCl silencing is effective under conditions of high $[Cl^-]_i$. Patch clamp recording of spontaneous activity of a $GluCl^+$ DRG while 20nM IVM is applied. Patch pipette was filled with an intracellular solution containing 80mM Cl^-. Under these recording conditions GluCl activation depolarizes the membrane potential, yet is still effective at reducing firing.

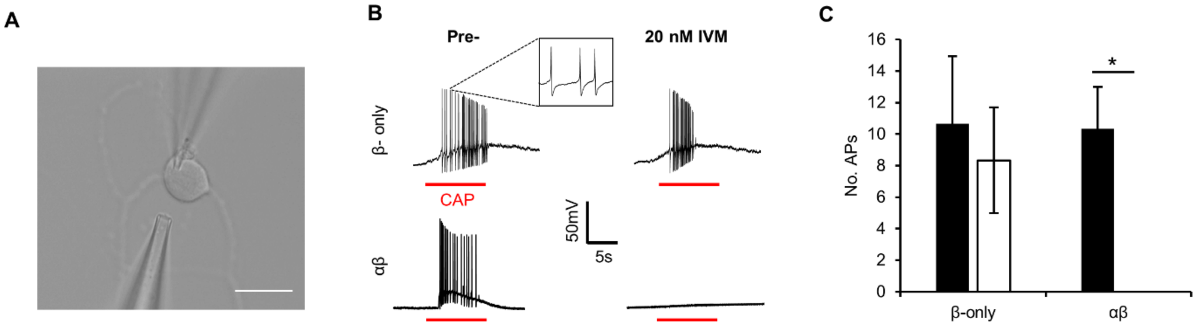

Supplementary Figure 3. GluCl activation prevents action potential firing in response to capsaicin. (**A**) Bright field image of experimental setup. Small diameter (15-25μM) neurons expressing GluCl were assessed for action potential firing during a 10s application of 1μM capsaicin (CAP) applied locally by a second pipette positioned adjacent to the cell. Scale bar represents 25μM. (**B**) Representative firing of $GluCl^+$ neurons in response to CAP, before and 15 mins after treatment with 20 nM IVM. (**C**) Quantification of action potentials fired in

response to CAP pre- and 15 mins following 20 nM IVM. $*P < 0.01$, Student's paired *t*-test comparing pre- and post-IVM responses ($n = 6$ for both groups). Data represents mean ± s.e.m.

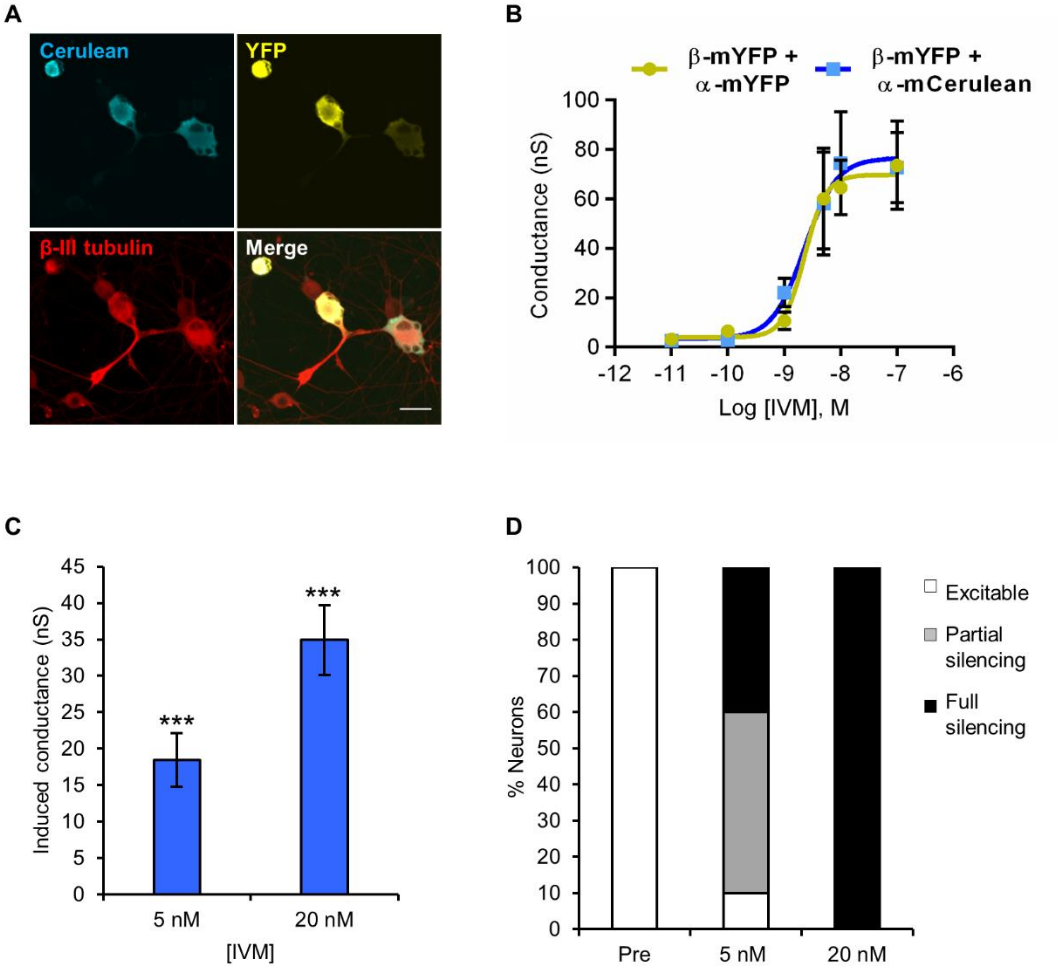

Supplementary Figure 4. Replacing YFP with Cerulean does not alter function of GluClα. (**A**) Endogenous cerulean and YFP signal in addition to immunostained β-III tubulin of DRG neurons electroporated with GluClα-mCerulean and GluClβ-mYFP. Scale bar, 30 μm. (**B**) Dose-response relation for IVM activation of GluCl expressed in HEK 293 cells. EC_{50} is 2.6 ± 0.2 nM for β-mYFP + α-mYFP and 2 ± 0.3 nM for β-mYFP + α-mCerulean expressing cells ($P > 0.99$, Student's unpaired *t*-test). Each data point represents the mean ± s.e.m. of the input conductance of at least 5 cells at each IVM concentration. (**C**) Membrane conductance induced

by acute treatment of $GluCl^+$ DRG with 5 and 20 nM IVM. ***$P < 0.001$, Student's paired *t*-test comparing pre- and post-IVM levels (5 nM $n = 10$ cells, 20 nM $n = 6$ cells.) Data represents mean ± s.e.m. (**D**) Excitability status before and after IVM treatment. 5 nM $n = 10$ cells, 20 nM $n = 6$ cells.

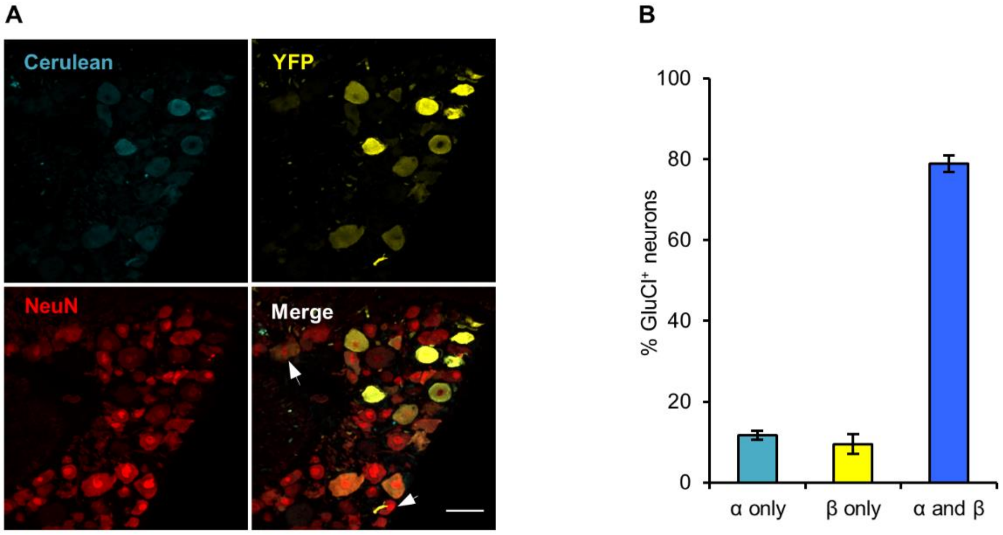

Supplementary Figure 5. GluClα and β subunits co-localize following i.t delivery. (**A**) L4 DRG 4-weeks post-AAV delivery imaged for native cerulean/YFP fluorescence and immunostained NeuN. White arrows mark neurons only expressing a single subunit. Scale, 50 μm. (**B**) Quantification of co-expression. Data represents the mean ± s.e.m from three animals (total= 323 cells).

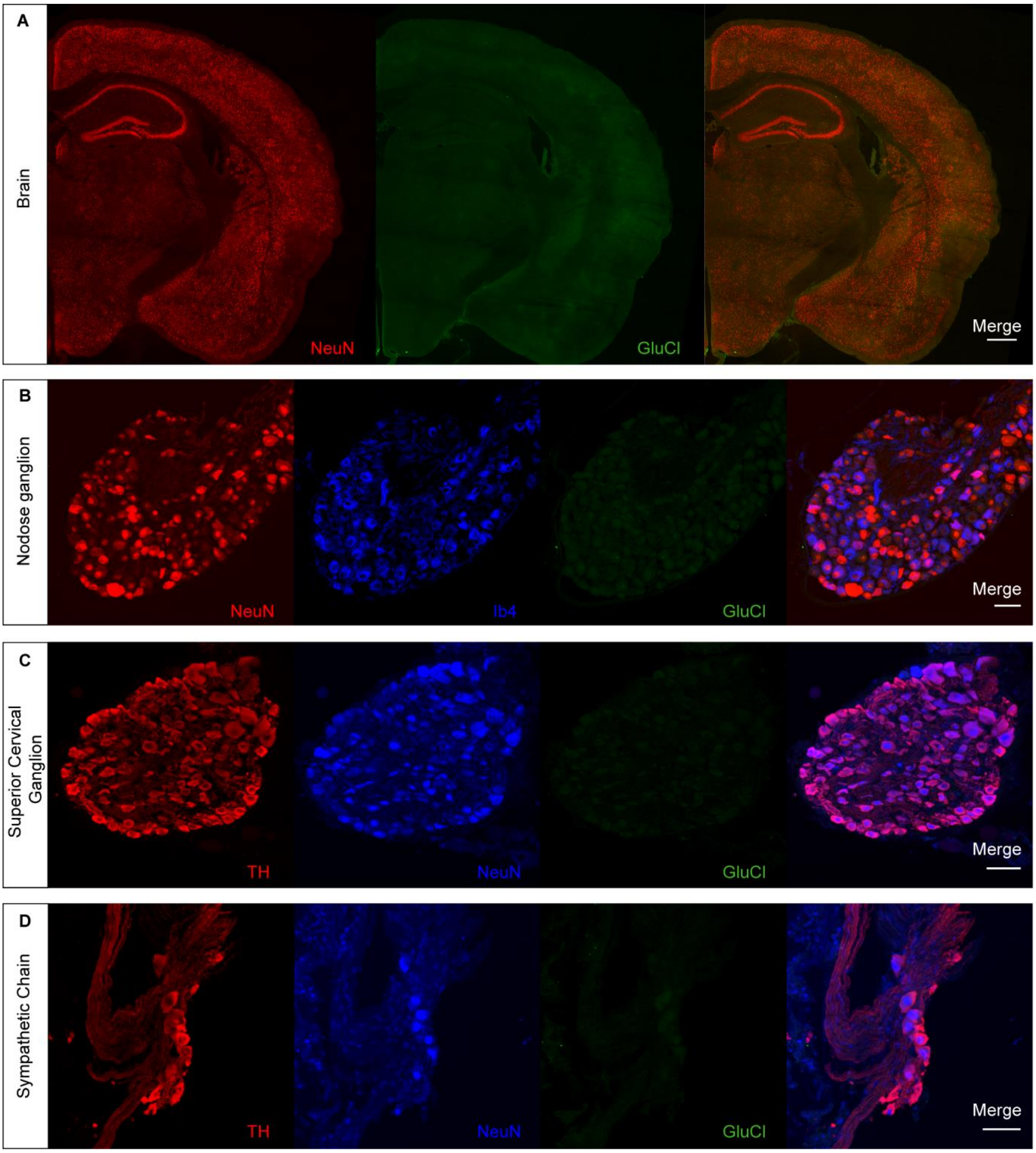

Supplementary Figure 6. GluCl expression is absent in brain and autonomic structures following i.t. AAV delivery. Whole brains and a range of ganglia were assessed from $GluCl^+$ animals following i.t. delivery of AAV-GluCl (n = 3). No GluCl expression was observed in (**A**) brain (scale bar 500µm), (**B**) nodose ganglia, (**C**) Superior cervical ganglion, or (**D**) lumbar sympathetic chain neurons. Scale bar represents 50µm unless stated otherwise. *TH-tyrosine hydroxylase*.

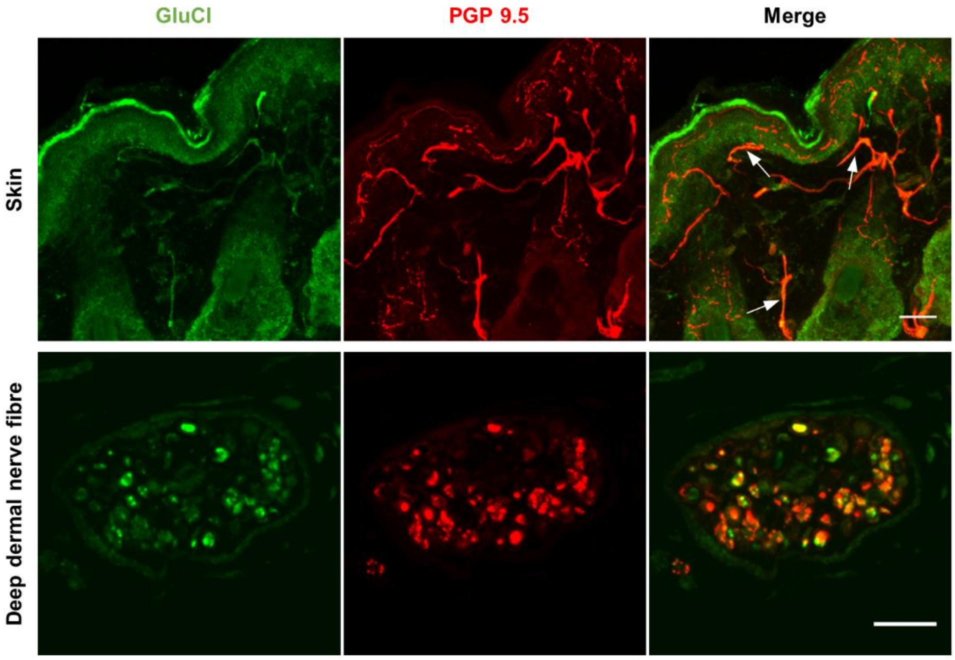

Supplementary Figure 7. GluCl$^+$ afferents in skin. Hind paw skin taken from GluCl AAV animals four weeks post AAV delivery. Representative images ($n = 3$ GluCl$^+$ animals) showing immunostaining for YFP (*green*) and PGP 9.5 (*red*) in the skin and a transverse section of a deep dermal nerve fibre. Scale bar, 20 µm.

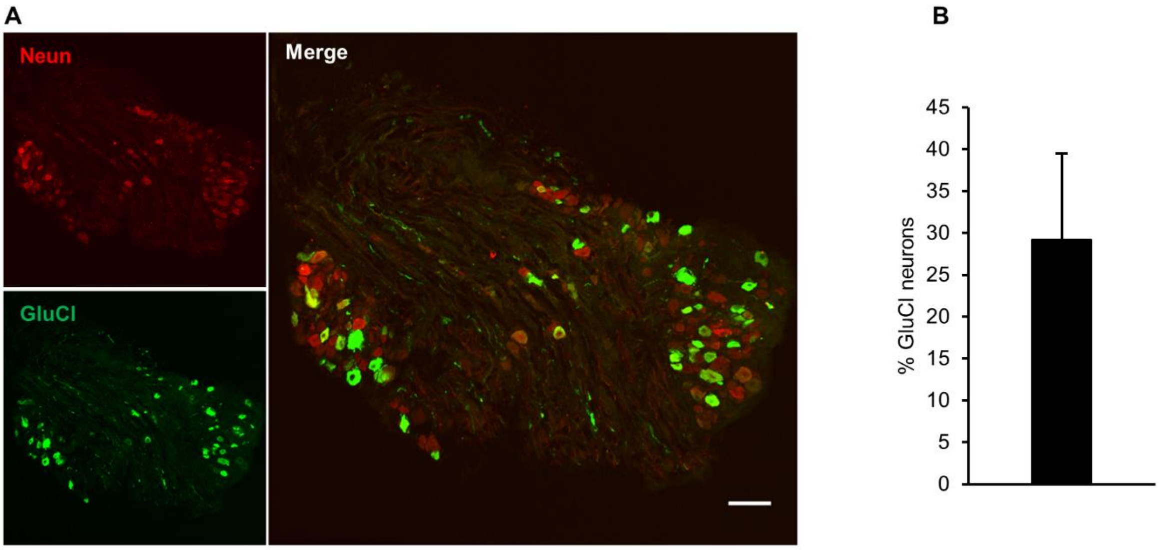

Supplementary Figure 8. Long-term expression of GluCl after a single i.t dose of GluClαβ-AAV. Anatomical analysis of DRG taken from animals 7 months post i.t. delivery. (**A**) L4 DRG immunostained for NeuN (*red*) and YFP (*green*). Scale bar, 100 μm. (**B**) Total neurons that are GluCl^{+}. Data represents mean ± s.e.m of four animals (with one animal from the cohort excluded for zero GluCl expression) and a total of 1762 cells.

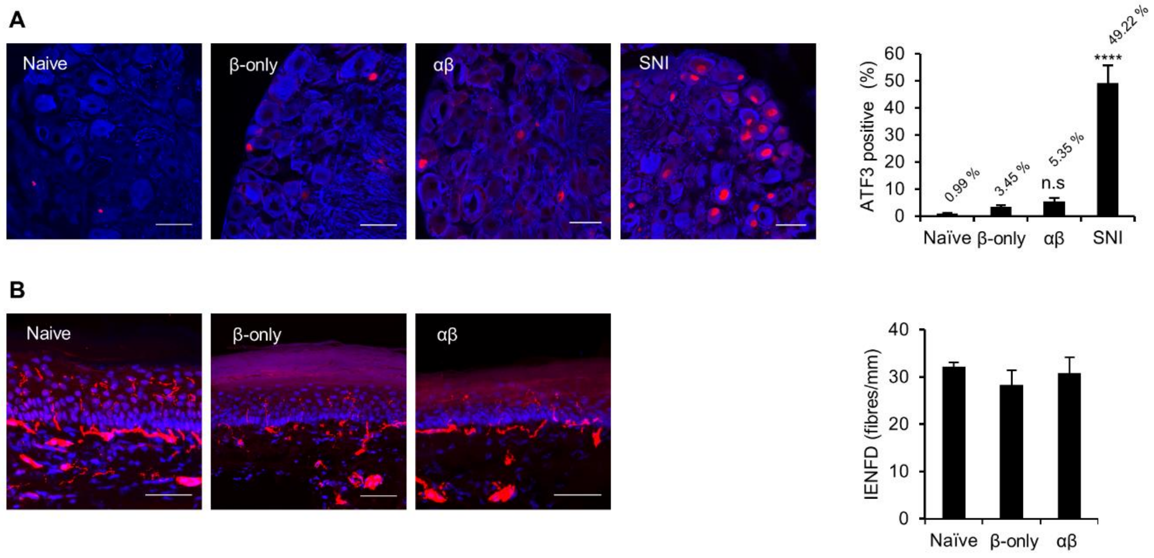

Supplementary Figure 9. DRG ATF-3 expression and cutaneous afferent innervation in aged GluCl-AAV animals. Tissue derived from animals 7-months following i.t. delivery of GluCl-AAV. Naïve group represents age matched animals that did not undergo i.t. surgery and SNI group represents animals 4-weeks post SNI as a positive control for nerve injury. (**A**) *Left*, Representative images of β-III tubulin (*blue*) and ATF-3 (*red*) staining of L4 DRG sections. *Right,* Quantification of ATF-3 expression as a percentage of total neurons. ****$P < 0.001$, one-way ANOVA followed by post-hoc Bonferroni test, data represents mean ± s.e.m. Naïve $n = 3$, β-only $n = 3$, αβ $n = 4$, SNI $n = 4$ animals. (**B**) *Left*, Representative images of DAPI (*blue*) and PGP9.5 (*red*) staining of hind paw glaborous skin of aged cohorts. *Right,* Quantification of intra-epidermal nerve fibre density (IENFD). $P > 0.05$, one-way ANOVA

followed by post-hoc Bonferroni test, data represents mean ± s.e.m. Naïve $n = 3$, β-only $n = 3$ and αβ $n = 4$ animals. Scale bars represent 50 µm.

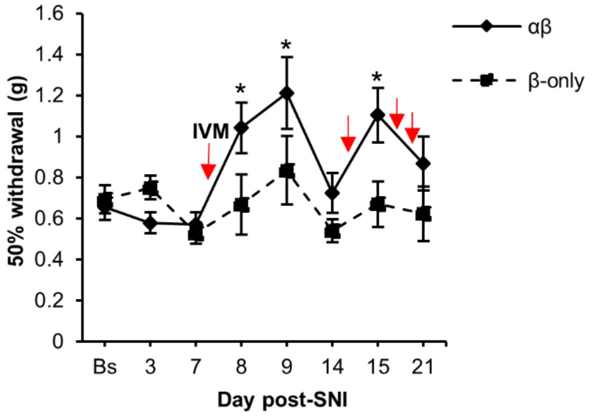

Supplementary Figure 10. Mechanical thresholds of the contralateral paw of GluCl^{+} animals following SNI. Time-course of contralateral paw mechanical sensitivity following SNI (related to **Fig 5A**) and dosing with IVM. Red arrows signal the time at which animals received a systemic dose of 5 mg/kg IVM. *$P < 0.05$, RM-ANOVA followed by post-hoc Bonferroni test comparing post-SNI time-points with baseline, $n = 11$ animals per group.
